# One-stage procedure with debridement and arthrodesis using external fixators for the treatment of chronic hindfoot infections: a retrospective study

**DOI:** 10.3389/fsurg.2026.1787590

**Published:** 2026-04-10

**Authors:** Ying Li, Ning Sun, Wenjing Li, Baozhou Zhang, Yong Wu, Hui Du

**Affiliations:** 1 Department of Foot and Ankle Surgery, Beijing JiShuiTan Hospital, Capital Medical University, Beijing, China; 2 Department of Foot and Ankle Surgery, Beijing JiShuiTan Hospital, The Fourth Clinical College of Peking University, Beijing, China

**Keywords:** arthrodesis, arthroscopy, chronic infections, hindfoot, one-stage procedure

## Abstract

**Background:**

Chronic hindfoot infections poses a significant threat to limb integrity and quality of life. The one-stage procedure, involving implants removal, debridement, and simultaneous arthrodesis, may serve as an alternative choice. The present retrospective study aimed to assess the efficacy of the one-stage procedure using external fixators for the treatment of chronic hindfoot infections.

**Methods:**

From April 2016 to December 2022, 21 patients with chronic hindfoot infections underwent one-stage procedure with debridement and arthrodesis using external fixators. Pain and joint function were evaluated using the Visual Analogue Scale (VAS) and American Orthopaedic Foot and Ankle Society (AOFAS) Score. Infection recurrence and osseous consolidation were appraised using the Musculoskeletal Infection Society criteria and radiological examination.

**Results:**

The tibiotalar, tibiotalocalcaneal, and pan-tarsal arthrodesis were performed in 6, 14 and 1 patients, respectively. The surgical debridement procedures were performed using arthroscopic (*n* = 9) and combined open (*n* = 12) methods. The mean follow-up duration was 38.2 (range 25–103) months. The procedure achieved significant pain relieve and functional improvement, as evidenced by the substantial improvement in VAS and AOFAS score from 6.4 ± 1.7 and 40.0 ± 11.6 preoperatively to 1.3 ± 1.4 (*p* < 0.001) and 78.4 ± 10.8 (*p* < 0.001) postoperatively, respectively. No instance of infection recurrence was observed during follow-up, and successful osseous consolidation was achieved in 20 patients.

**Conclusions:**

The one-stage procedure with debridement and arthrodesis using external fixators represented an alternative method for the treatment of chronic hindfoot infections.

## Background

Septic arthritis of the hindfoot presents a severe clinical challenge, often stemming from hematogenous dissemination from other infection sites, direct inoculation due to trauma, and contiguous spread from neighboring tissues (1, 2). Predominantly, *Staphylococcus aureus* and *Staphylococcus epidermidis* are the primary causative bacteria (3, 4). Inadequate intervention for septic arthritis of hindfoot can lead to irreversible articular surface damage, infective synovitis, and osteomyelitis, culminating in chronic pain, deformity, and joint dysfunction, significantly impacting patients' quality of life. The primary treatment objectives encompass infection control, adequate soft tissue coverage, restoration of limb length, and joint arthrodesis.

For individuals afflicted with chronic hindfoot infections, debridement and arthrodesis are often indispensable to restore hindfoot stability and enable painless weight-bearing. Thorough debridement preceding ankle arthrodesis is imperative. The conventional two-stage procedure is a widely recommended approach for managing chronic hindfoot infections, involving implants removal, debridement, spacer placement, antibiotic therapy, and subsequent second-stage arthrodesis (4–6). However, the second-stage surgery may be intolerable for patients with significant comorbidities, such as cardio-cerebrovascular disease, peripheral vascular disease, and severe soft tissue conditions (7, 8). The one-stage procedure, involving implants removal, debridement, and simultaneous arthrodesis, may serve as an alternative choice to mitigate complications and mortality (9–11). Thorough debridement of the articular cavity is particularly crucial, especially for the one-stage procedure, preventing hidden contaminants in crevices from becoming the primary instigators of infection recurrence. Some studies stated that the arthroscopic debridement, offering an optimal visual field for joint cavity cleansing, is considered an effective method for treating septic ankle conditions, yielding favorable clinical outcomes and fewer complications (12, 13).

Attaining successful arthrodesis remains a viable option, even in the presence of active infection, serving as a final recourse to salvage the limb from amputation. To achieve satisfactory osseous fusion, stable fixation and favorable soft tissue conditions are imperative (7, 14–16). Chronic infection heightens the risk of bone nonunion, prompting a preference for external fixation in such conditions (7, 15, 17, 18). External fixation offers numerous advantages, including soft tissue protection, reduced metal residue, interfragmentary compression, and gradual correction of deformities (19–21). While some studies have explored the use of internal fixation or a combination of internal and external fixation for arthrodesis in patients with septic arthritis, achieving satisfactory clinical outcomes, the associated risks of implant loosening, diminished bone stock, and residual foreign bodies are inevitable with internal fixation in hindfoot arthrodesis for patients with chronic infection (14, 18, 22). In the context of the one-stage procedure with debridement and arthrodesis, external fixation continues to be the preferred choice to minimize the risk of infection recurrence (21, 23). Therefore, the present study aimed to explore the outcomes of the one-stage procedure involving debridement and arthrodesis using external fixators for the treatment of chronic hindfoot infections.

## Methods

### Patients

A retrospective review of medical records (April 2016–December 2022) at our institution involved patients diagnosed with chronic hindfoot infections, characterized by septic arthritis of the ankle and/or subtalar joints affecting the calcaneus, talus, navicular, cuboid, associated joints, and surrounding soft tissues. Diagnosis was confirmed through clinical, radiological, and microbial culture. Data including etiological antecedents, comorbidities, clinical symptoms, laboratory and radiological examination results, surgical procedures, postoperative management, and outcomes were recorded and analyzed.

### Surgical intervention

**Figure 1:**
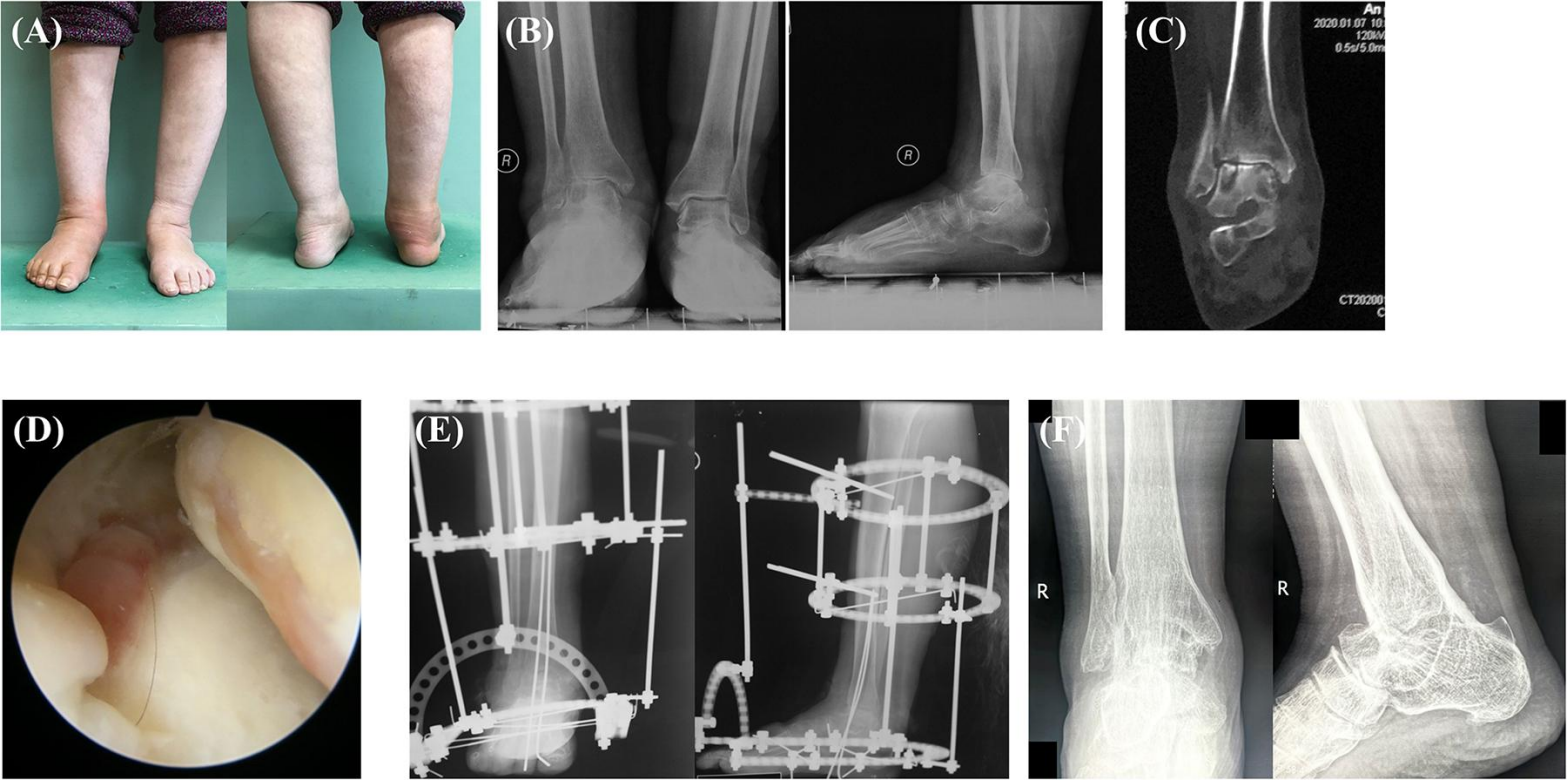
A 75-year-old female patient presented with a three-year history of inexplicable swelling and pain in the right ankle joint, with exacerbation over the past year characterized by swelling, warmth, and nocturnal pain **(A)**. Preoperative x-ray **(B)** and CT **(C)** imaging revealed joint damage and arthritic changes in the ankle and subtalar joints. Arthroscopy-assisted right tibiotalocalcaneal debridement and arthrodesis was performed. We found the cartilage detachment and synovial hyperplasia during surgery **(D)**. Postoperative x-ray assessment demonstrated favorable fusion positioning and proper hindfoot alignment **(E)** At final follow-up, the satisfactory osseous consolidation and hindfoot alignment were observed **(F)**.

**Figure 2:**
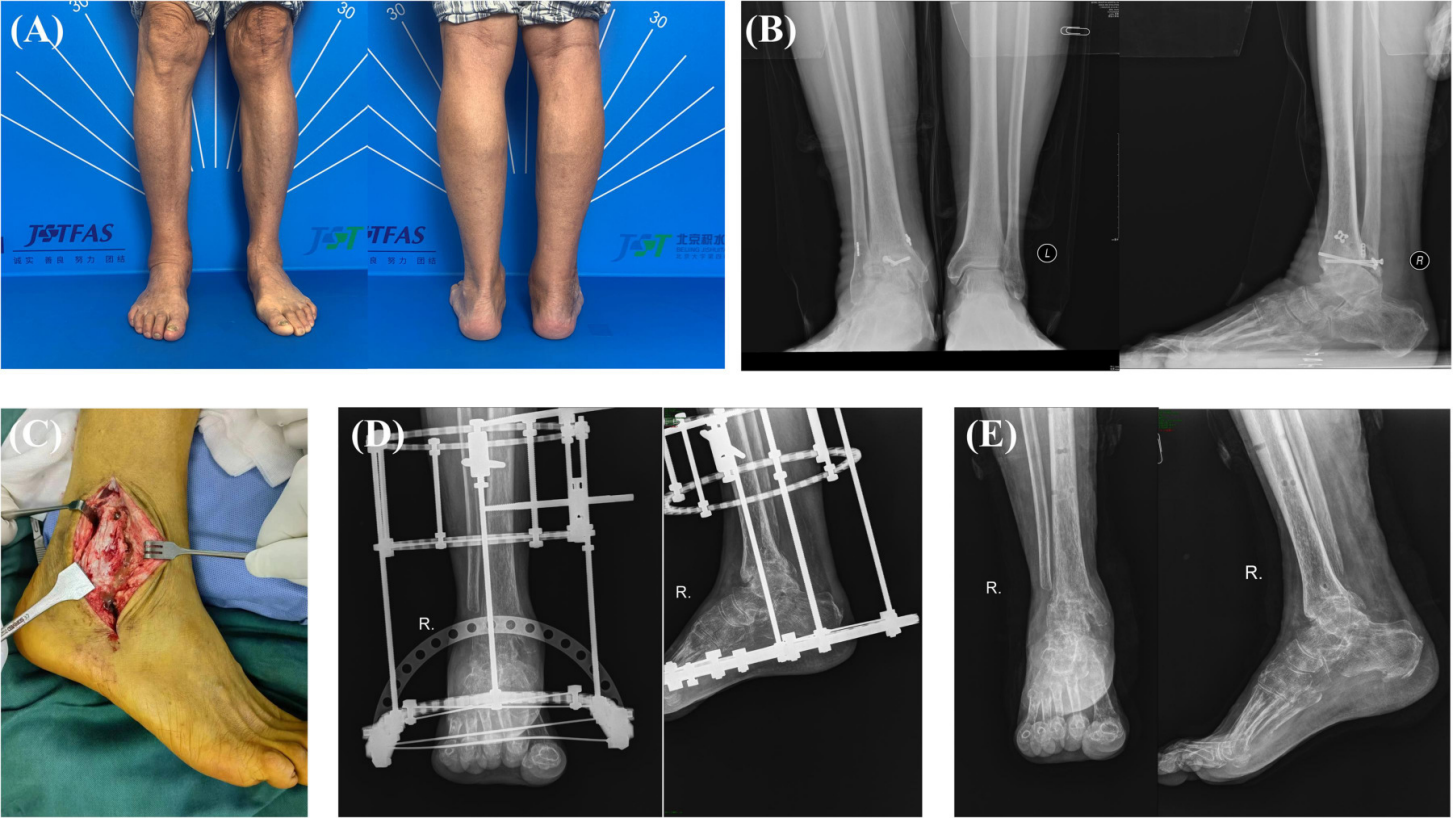
A 77-year-old male patient presented with chronic hindfoot infections subsequent to surgical intervention for right ankle fractures. The patient reported persistent pain and swelling in the right ankle, with acceptable hindfoot alignment **(A)**. Preoperative weight-bearing x-ray imaging revealed joint destruction in the right ankle **(B)**. Intraoperatively, purulent accumulation was noted within the right ankle joint **(C)**. Postoperatively, x-ray evaluation demonstrated favorable fusion positioning and proper hindfoot alignment **(D)**. At the three-month follow-up, satisfactory osseous consolidation was observed at the fusion site subsequent to the removal of external fixators **(E)**.

The surgical procedures encompassed thorough debridement and bone fusion with stable fixation. The surgical approach should be as consistent as possible with the previous approach. The anterolateral approach was more ideal for dealing with the ankle and subtalar joint. Bacterial cultures were collected before debridement, with five specimens of infected tissues submitted for microbiological analysis. Debridement involved complete removal of implants, infected and necrotic tissue, and scarring to healthy tissue areas. The debridement of hindfoot was performed under both arthroscopy (Figure 1) and combined open (Figure 2) methods. Arthroscopic debridement was prioritized due to its efficacy in removing infected tissue, contaminated cartilage, and intra-articular foreign-bodies while minimizing soft tissue trauma. Moreover, the combined anterior-posterior arthroscopic approach enabled more extensive debridement of periarticular infected and necrotic tissues than single approach. The open anterior-posterior approach was usually avoided due to concerns regarding excessive soft tissue disruption. However, in cases of extensive and severe infection, arthroscopy alone was insufficient for thorough debridement, necessitating combination with open procedures. For patients with previously removed internal fixation devices, the arthroscopic approach was the preferred choice. While, for patients with residual internal fixators, a combined arthroscopic and open approach was usually unavoidable. The reserved tissue exhibited normal color, texture, and the absence of purulence, indicating thorough debridement.

Our study included patients who underwent three arthrodesis techniques: tibiotalar in 6 cases, tibiotalocalcaneal in 14 cases, and pan-tarsal in 1 case. For tibiotalar arthrodesis, the cartilage of the ankle was removed in debridement process. Following exposure of healthy trabecular bone, temporary fixation with Kirschner wires was performed. Intraoperative fluoroscopy confirmed favorable fusion positioning. The ankle was usually fixed in a neutral position, with slight external rotation of the foot relative to the tibia (5–10 degrees), slight outward rotation of the hindfoot (5–7 degrees), slight backward movement of the talus relative to the tibia, and no inward or outward movement of the talus in the ankle mortise. The arthrodesis was achieved using the Ilizarov circular fixator (Double Medical, Fujian, China). The installation of external fixators adhered to established principles for arthrodesis. The foot and tibia were overall compressed and fixed by the external fixator device. For tibiotalocalcaneal arthrodesis, the cartilage of ankle and subtalar joints should be removed. In addition to the external fixation device, two Kirschner wires were inserted from the plantar surface into the medullary cavity of the tibia to fix both the ankle and subtalar joints simultaneously. In our study, one patient underwent pan-talar arthrodesis for septic ankle and subtalar arthritis secondary to trauma, complicated by preexisting rheumatoid arthritis. The surface of ankle, subtalar, talonavicular, and calcaneocuboid joints were clean, and the joints were fixed and compressed by external fixator and Kirschner wires.

### Perioperative management

All patients received preoperative and postoperative antibiotic therapy for a minimum of 6 weeks. Upon detection of bacterial pathogens, targeted antibiotics were immediately administered. For patients with severe osteomyelitis or joint infection, personalized treatment plans were developed based on clinical symptoms and infection indicators. Meticulous pin care was conducted to prevent pin tract infections. The frequency of cleaning the pin site should be related to the local condition around the pin tract. If swelling, crusts, or signs of exudate were observed, the pin site should be cleaned more frequently (1–2 times a day). Once the pin site healed with clean and dry status, the cleaning frequency could be reduced. If there was exudate, the sterile gauze should be placed over the pins. Patients began partial weight-bearing 3 weeks postoperatively and achieved full weight-bearing at 12 weeks. External fixators were removed 12 weeks postoperatively, following confirmation of reliable arthrodesis by CT examination. Additionally, 2–3 weeks prior to external fixator removal, the screws of the bolt connecting the tibial ring and the foot ring were intentionally loosened to increase stress at the joint fusion region, promoting bone fusion and remodeling.

### Evaluation

The Visual Analog Scale (VAS) was used to assess pain improvement, the American Orthopaedic Foot and Ankle Society Score (AOFAS score) was employed to evaluate joint functional status, and the Musculoskeletal Infection Society (MSIS) criteria were used to assess postoperative infection recurrence and the effectiveness of infection control. Radiological examinations were used to evaluate osseous consolidation, with fusion determined by the presence of complete cortical bridging in three cortices (anterior, posterior, medial, and lateral) without a fracture line. The time of external fixator removal, the maximum distance and time of walking, postoperative complications were recorded.

### Statistical analysis

Statistical analyses were conducted using SPSS software (version 19.0; IBM Corp, Armonk, NY). Normal distribution was evaluated using the Kolmogorov–Smirnov test. Differences between preoperative and postoperative VAS scale and AOFAS score were determined using the paired *t*-test, with *p* values < .05 considered statistically significant.

## Results

### Patients characteristics

Table 1 presents the demographic and clinical characteristics of the 21 patients included in present study. The cohort comprised 9 females and 12 males, with a mean age of 52.6 years and an average follow-up duration of 38.2 months (range 25–103 months). The mean BMI was 27.2 kg/m^2^. The antecedents of chronic hindfoot infections included invasive surgery after trauma in 16 patients, idiopathic infection in 4 patients, and local injection in 1 patient. Septic ankle arthritis occurred in all cases, with concomitant septic subtalar arthritis in 15 cases. Pain was the most common symptom, with swelling, deformity, instability, purulent discharge, and raised skin temperature also reported. Six patients had comorbid type 2 diabetes. Laboratory findings revealed increased white blood cells, neutrophils, C-reactive protein, and erythrocyte sedimentation rate, with positive bacterial cultures observed in 13 cases, including *Staphylococcus aureus*, *Staphylococcus epidermidis*, *Bacillus subtilis*, and *Sphingobacterium spiritivorum*.

**Table 1:** Patients' demographics and characteristics.

Patients (*n* = 21)	Data	Number	Percentage
Gender	Female	9	
	Male	12	
Age (y)		52.6 ± 16.0 (range 21–77)	
Body mass index (kg/m^2^)		27.2 ± 4.1 (range 20.3–37.7)	
Antecedents	Invasive procedures after trauma	16	76.20%
	Idiopathic infection	4	19.00%
	Local injection	1	4.80%
Affected side	Right	13	61.90%
	Left	8	38.10%
Symptoms	Pain	21	100.00%
	Swelling	19	90.50%
	Deformity	18	85.70%
	Instability	12	57.10%
	Purulent discharge	12	57.10%
	Raised skin temperature	6	28.60%
Bacterial culture	Positive results	13	
	*Staphylococcus aureus*	9	69.23%
	*Staphylococcus epidermidis*	2	15.38%
	*Bacillus subtilis*	1	7.69%
	*Sphingobacterium spiritivorum*	1	7.69%

### Outcomes

The tibiotalar, tibiotalocalcaneal, and pan-tarsal arthrodesis were performed in 6, 14 and 1 patients, respectively. The surgical debridement procedures using arthroscopic (*n* = 9) and combined open (*n* = 12) methods. The patients actually removed the external fixation device on average 4.2 months after the operation and began full weight-bearing activities. At final follow-up, both pain and functional assessment scores demonstrated significant improvement compared to preoperative baselines (Table 2). The VAS scores decreased from 6.4 ± 1.7 preoperatively to 1.3 ± 1.4 postoperatively (*p* < 0.001), representing an exceptional magnitude of pain reduction. The AOFAS score increased significantly from 40.0 ± 11.6 to 78.4 ± 10.8 (*p* < 0.001), demonstrating a clinically meaningful improvement in hindfoot function. No infection recurrence was observed, and osseous consolidation was achieved in 20 patients. Bone nonunion was detected in one patient by CT scan. The patient reported occasional pain without any other discomfort. The patient's daily life had improved significantly postoperatively, leading to refusal of further intervention. No fractures were identified after external fixator removal. Six patients reported nearly normal walking time and distance, while the maximum walking time and distance for other patients averaged 23.3 ± 10.1 min and 1,008.3 ± 782.1 meters. The most common postoperative complications included pin tract infection in 6 patients and toe numbness in 4 patients. Among the 5 patients with postoperative pin tract infection, successful resolution was achieved through dressing and anti-infection drugs, while 1 patient required debridement to address infectious and necrotic tissues. We found that the infections localized around the pin tract and had not spread to the fusion area during surgery.

**Table 2:** Clinical outcomes after operation.

Evaluation	Preoperation	At final follow-up	*P* value
Visual analogue Scale	6.4 ± 1.7	1.3 ± 1.4	<0.001
AOFAS Score	40.0 ± 11.6	78.4 ± 10.8	<0.001
Recurrence of infection		0 (0%)	
Osseous consolidation		20 (95.2%)	

AOFAS Score, American orthopaedic foot and ankle society score.

## Discussion

Chronic hindfoot infections typically results from microbial infections entering the joint through wounds, invasive surgeries, or from other infected areas in the body. Symptoms may include persistent pain, swelling, warmth, redness, and limited joint motion. Left untreated, chronic hindfoot infections can lead to joint damage, bone erosion, and systemic complications. Early and prompt treatment are crucial to prevent long-term joint damage and complications. Debridement and arthrodesis are the primary surgical methods for treating chronic hindfoot infections.

Thorough debridement is a prerequisite for successful bone fusion. Intra-articular debridement has always been a challenge in surgery due to the narrow joint space of the hindfoot. Pollutants hidden in the gaps may become the main culprits for infection recurrence. In our clinical practice, we attempted to use arthroscopy for joint decompression, infectious nidus evacuation, and intra-articular lavage. Arthroscopy often achieved precise debridement of the intra-articular space and minimal periarticular soft tissue invasion (12, 13). When necessary, a combined anterior and posterior arthroscopic approach can be used to more extensively clean the infected and necrotic tissues around the joint. In contrast, a combined anterior and posterior open approach to clean the infection may cause significant damage of soft tissues. However, for patients with severe and extensive infections, especially those who need to remove internal fixation devices, combined open debridement is inevitable. Even in this situation, the combined use of arthroscopy could still improve debridement efficiency, reduce soft tissue trauma, and help treat joint cavity lesions. The outcomes of our study presented that the infection of hindfoot was well controlled after surgery. No recurrence of infection was found in 21 patients at the final follow-up. Compared with similar studies (14, 18, 22–25), our study demonstrated satisfactory infection control outcomes. The absence of septic arthritis recurrence in all patients demonstrated that arthroscopic debridement may be an alternative method for treating chronic hindfoot infections.

The external fixation can minimize implant-related tissue infection recurrence, early loosening, implant failure, and soft tissue damage to the greatest extent possible, compared to internal fixation. While the rigid internal fixation provides immediate stability that favors bone fusion, the Ilizarov external fixator offers comparable osseous integration through an adjustable dynamic compression method. In our clinical practice, 2–3 weeks prior to external fixator removal, the screws of the bolt connecting the tibial ring and the foot ring were intentionally loosened to increase stress at the joint fusion region, promoting bone fusion and remodeling. In our study, bone healing was achieved in 95.2% of the cases, showing a similar bone fusion rate to other reports (14, 18, 22–25). Simoni et al. treated 52 patients with septic osteoarthritis of the ankle through a two-stage operation, using extramedullary internal fixation for arthrodesis and reporting a nonunion rate of only 8.75% (24). Kollig et al. performed 15 arthrodeses to treat septic ankle using hybrid external fixation, achieving solid tibiotalar fusion in 12 patients without fistula and in 2 patients with fistula (18). They suggested that the one-stage procedure and hybrid external fixation provided a successful alternative for treating septic ankle. One patient in our study suffered bone non-union, related with the high risk factors of uncontrolled diabetes and related complications. The patient reported no obvious ankle discomfort except for occasional pain and significant improvement of foot function in daily life. The patient expressed satisfaction with the postoperative outcomes and refused further surgical intervention. We believe that external fixation is a favorable method for treating chronic hindfoot infections, especially for one-stage procedure, however, the potential shortcomings of external fixation, such as the risk of pin tract infection or loosening, discomfort, and movement difficulty, should not be ignored.

Conventionally, two-stage procedure (implant removal, debridement, spacer placement, and delayed arthrodesis) has been favored for chronic infections of hindfoot to ensure thorough infection control. However, this method has significant shortcomings. Prolonged treatment increases risks for patients with comorbidities, such as diabetes and cardiovascular disease. Prolonged immobilization and spacer-related discomfort significantly impair patients' quality of life. Our study supports the one-stage procedure as an alternative method. Our results demonstrated significant improvements in infection control, bone fusion, and functional outcomes at final follow-up, consistent with outcomes of other studies for one-stage and two-stage procedures (14, 18, 22–25). Hartmann et al. conducted a study comparing infection control, ankle function, and bone consolidation between two-stage and one-stage procedures in patients with hindfoot and ankle infections (23). They found no significant differences in infection control, AOFAS score, and radiologic consolidation between the two groups. From our experience, thorough debridement and reliable fixation are the necessary conditions for achieving one-stage procedure. We believed that the controlling infection and promoting bone fusion are mutually reinforcing. Thorough debridement and removal of necrotic and infected tissues can control infection and create better conditions for bone healing. Simultaneously, joint fusion requires sufficient contact between joint surfaces, which can eliminate dead spaces and facilitate infection control.

Postoperative management of patients is crucial for successful treatment. The postoperative pin tract infection was the most common complication in our study. Six patients suffered pin tract infections, which were successfully resolved through dressing changes, debridement, and anti-infection treatment, without causing more serious consequences. Meticulous pin care should be conducted to prevent pin tract infections. There is no consensus on the best nursing care for pin tract. Considering the patient's previous severe joint infection, we preferred a more aggressive nursing approach. Pin tract cleaning frequency should depend on local conditions. The pin site should be cleaned 1–2 times a day or more, if swelling, crusts, or signs of exudate were observed. Once the pin site healed with clean and dry status, the cleaning frequency could be reduced. The postoperative weight-bearing also needs to be taken into consideration. The patients were asked to begin partial weight-bearing 3 weeks after the operation. The weight-bearing was gradually increased, and full weight-bearing was achieved at 12 weeks. After the reliability of bone fusion was confirmed by imaging examinations, the external fixator was removed. Subsequently, the patients could engage in gradually increasing weight-bearing activities until normal walking was possible.

This study has several limitations. Firstly, the small sample size limited the statistical power for identifying significant associations between variables. In further research, we will expand enrollment to improve robustness of the analysis. Secondly, due to the limited number of cases at our hospital, patients who had two-stage procedure or internal fixation were excluded. In further study, we will recruit more patients for conducting subgroup comparative analyses. Thirdly, as a retrospective study, it is hard to fully avoid bias from data inconsistencies and methodological variations in research process. We implemented standardized data collection protocols, with predefined sources and recording procedures to ensure accuracy. Uniform methods of analysis were applied across all cases to minimize variability.

## Conclusions

In conclusion, chronic hindfoot infections present significant challenges, often requiring surgical intervention to control infection and achieve arthrodesis. Our study confirmed the feasibility and acceptable outcomes of one-stage procedure with debridement and arthrodesis using external fixators in the treatment of chronic hindfoot infections.

## Data Availability

The datasets used in this study are available from the corresponding author upon request.
